# P04 Introduction of a multidisciplinary tool to switch from IV to oral antibiotics

**DOI:** 10.1093/jacamr/dlad143.008

**Published:** 2024-01-03

**Authors:** Laura Ahearn, Andrew Stone, Hou Law

**Affiliations:** The Royal Oldham Hospital, Oldham, UK; The Royal Oldham Hospital, Oldham, UK; The Royal Oldham Hospital, Oldham, UK

## Abstract

**Background:**

Appropriate and timely switching from IV to oral antibiotic therapy has been a long standing problem in hospitals. We aim to improve this at a busy district general hospital through the adoption of a decision tool developed from guidance from Public Health England ‘Start Smart – then Focus toolkit’ (Public Health England, March 2015) and ‘Antimicrobial intravenous-to-oral switch: criteria for early switch’ (gov.uk).

**Baseline audit:**

To audit local practice, a case note review of 10 current inpatients on two respiratory/general medical wards who had been on IV antibiotics for 48 h or more was performed in August 2023. All patients had received clinician review within the past 24 h; none had received a formal review in keeping with the NHS England guidance IV-to-oral switch (IVOS) decision aid. The time on IV antibiotics ranged from 3 days to 11 days. Six patients (60%) met the criteria for oral switch but remained on IV antibiotics, a further one patient who met criteria was kept on IV antibiotics as per microbiology advice. Two patients remained on IV antibiotics awaiting blood test results.

**Interventions:**

On the same wards, the following interventions occurred in August 2023: (i) agreement of local trial with 3 respiratory consultants and 2 ward managers; (ii) face-to-face teaching including pre- and post-knowledge tests with nursing staff (including night nurses)—9 sessions lasting 30 min each; (iii) attended board rounds to speak to the medical team regarding the implementation of a new tool; (iv) poster promotion; and (v) use of a protocol in the form of a sticker to be inserted into the clinical notes. Although any member of the multidisciplinary team (MDT) could complete the protocol, the aim was that night nurses would complete at least parts A, B and C.

**Reaudit:**

A similar case note review of 15 patients performed the week after the above interventions revealed all patients had now received a formal clinician review of their IV antibiotics within the past 24 h, in keeping with NHS England guidance IVOS decision aid. 10 patients had switched from IV to oral antibiotics, 1 was stopped and sent home the same day, 1 met the criteria but no rationale was written so unsure why IV antibiotics was continued and 1 was waiting for blood test results, 1 was kept on IV as per micro and 1 was awaiting CRP and WCC results.

**Results:**

The education sessions revealed major gaps in nursing knowledge about antimicrobial stewardship and their role. The protocol was quick to complete, and it was reported ticking mainly yes was a good way to identify patients suitable for switching. Some nurses stated the tool empowered them, allowing them to make a professional challenge for switch. Prescribers rationalizing their decisions prompted more IV to oral switch. Not all doctors liked completing the tool due to time or preference. Some nurses left out some fields of the protocol. Some nurses who had received training were happy to complete the question on blood tests, but others did not feel comfortable. Feedback from nurses was that where they completed the tool and the prescriber/clinician then did not complete the rationale box at the bottom, they felt deflated and not as motivated to complete the tool on further occasions.

**Conclusions:**

Interventions including education and the introduction of a sticker-based protocol were associated with a significant increase in IV to oral switch in antibiotics. Education revealed significant gaps in some nurses’ knowledge and their role in antimicrobial stewardship. Although the process was acceptable to many clinicians, there was not full engagement. For these reasons it is necessary to apply an MDT approach to antimicrobial stewardship. We need to be aware that different hospitals have different challenges and may require alternate approaches.

